# A metabolomic study on early detection of steroid-induced avascular necrosis of the femoral head

**DOI:** 10.18632/oncotarget.24150

**Published:** 2018-01-10

**Authors:** Xiangnan Ren, Wu Fan, Zixing Shao, Kaiyun Chen, Xuefeng Yu, Qionglin Liang

**Affiliations:** ^1^ Key Laboratory of Bioorganic Phosphorus Chemistry and Chemical Biology (Ministry of Education), Department of Chemistry, Tsinghua University, Beijing 100084, China; ^2^ The Fourth Affiliated Hospital, Nanchang University, Nanchang 330003, China

**Keywords:** metabolomics, lipid metabolism, femoral head necrosis, steroid-induced, early diagnosis

## Abstract

The early and accurate diagnosis of steroid-induced avascular necrosis of the femoral head (SANFH) is appealing considering its irreversible progression and serious consequence for the patients. The purpose of this study was to investigate the metabolic change of SANFH for its early detection. Two stages were designed in this study, namely discovery and verification. Except the biochemical index anomaly and the accidental death, 30 adult healthy adult Japanese white rabbits were used for screening out the potential metabolites in discovery experiment and 13 rabbits were used in verification experiment. The femoral heads were assessed with magnetic resonance imaging and transmission electron microscopy. The metabolomic profiling of serum samples were analysis by UHPLC-MS/MS. Metabolomic cluster analysis enable us to differentiate the rabbits without and with injection of the glucocorticoid in 1 week even when there is no obvious abnormal symptom in behaviors or imaging diagnosis. The majority of differential metabolites were identified as phospholipids which were observed significant change after injection of glucocorticoid in 1, 2, 3 weeks. And the results obtained in verification experiment of 6 weeks showed that these differential metabolites exhibited consistent trends in late progression with that in early-stage. At the end of 6 weeks the damage of SANFH could be verified by pathological imaging. Therefore the finding of serum metabolite profile links to the progression of SANFH and provides the potential of early detection of SANFH.

## INTRODUCTION

Using hormone continuously for a long time may lead to femoral head necrosis [1]. The glucocorticoid is one of the steroid and plays an important role on clinical treatment of organ grafting, systemic lupus erythematosus, rheumatic arthritis, dermatomyositis, myasthenia, but long time or excess use of the glucocorticoid could result in escalation of osteonecrosis [2–5]. Since the severe acute respiratory syndromes (SARS) outbreak in 2003, researchers have been paid more attention to steroid-associated osteonecrosis. It was irreversible once the symptom of steroid-induced avascular necrosis of the femoral head (SANFH) appeared. Therefore development of approaches or tools for early and accurate diagnosis of SANFH was appealing for its early prevent and treatment.

In present clinical research, the diagnosis was hysteretic and the prevention and cure of SANFH was lack of holism. Researchers generally focused on the part pathologic change. The prevention started after emergence of change on histopathology and imaging of part head of femur. In clinical diagnosis, the features of the SANFH patients were not obvious. The histopathology was obvious when the patients had already appeared the serious necrosis of the femoral head. At present, early diagnosis of SANFH depended on magnetic resonance imaging (MRI) scan [6]. However, the MRI only diagnose osteonecrosis after fat cell death 12–48 hours, when some pathologic changes were already generated, such as structural disorder of bone trabecula, pimelosis of marrow cavity, fatty degeneration and necrosis of osteocyte and so on [7–9]. Hence, it was meaningful to screen out risk factors of femoral head necrosis, monitor and intervene as early as possible, and prevent the emergence of early pathologic changes.

Metabolomics involves the analysis of entire pattern of low molecular weight compounds of endogenous or exogenous metabolites in biological samples, owing to pathophysiological stimuli or genetic modification [10]. Recent reports have suggested that metabolic profiles may reveal novel characteristics of metabolic abnormalities and improve the effect of diagnosis using LC-MS [11–19]. Especially for untargeted metabolite analysis, ultra-high performance liquid chromatography tandem mass spectrometry analysis (UHPLC-MS/MS) could obtain the comprehensive metabolic profiles, which contribute to discover new potential biomarkers, explore the mechanism of diseases and intervene as early as possible [20–29]. However, the diagnosis of SANFH by metabolomics has not been reported.

In this study, metabonomics study based on UHPLC-MS/MS was applied to investigate the serum metabolite profiling of rabbits of SANFH induced by prednisolone acetate, a kind of glucocorticoid. Potential biomarkers related to SANFH were discovered and verified, and their metabolic pathways were also discussed. Furthermore, clinical biochemistry, nuclear magnetism imaging and transmission electron microscopy (TEM) were also carried out for the phenotype proof of SANFH rabbits.

## RESULTS

**Figure 1:**
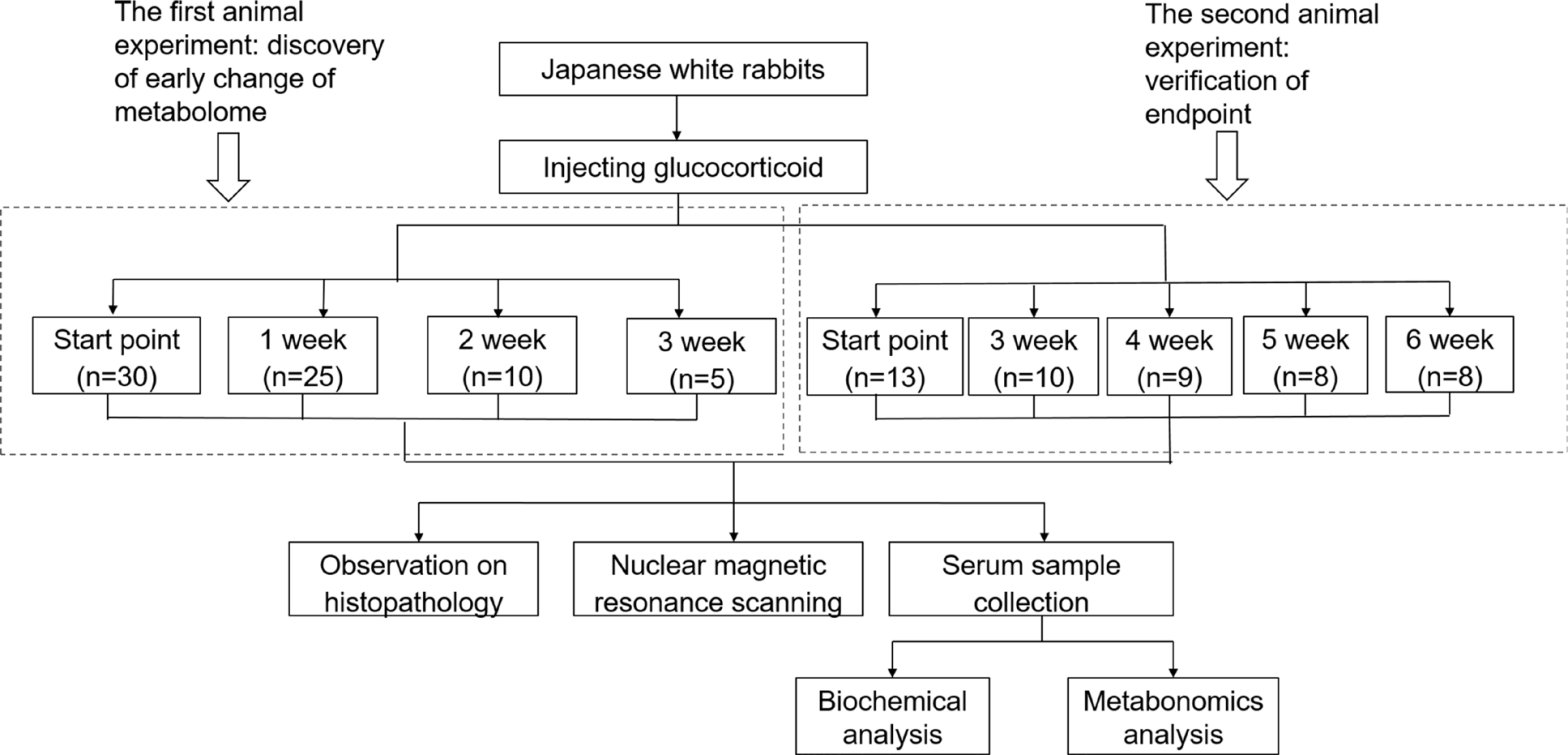
UPLC-MS/MS based roadmap for the serum metabolomics study on toxicity-attenuation effect of glucocorticoid injections in Japanese white rabbits

**Figure 2:**
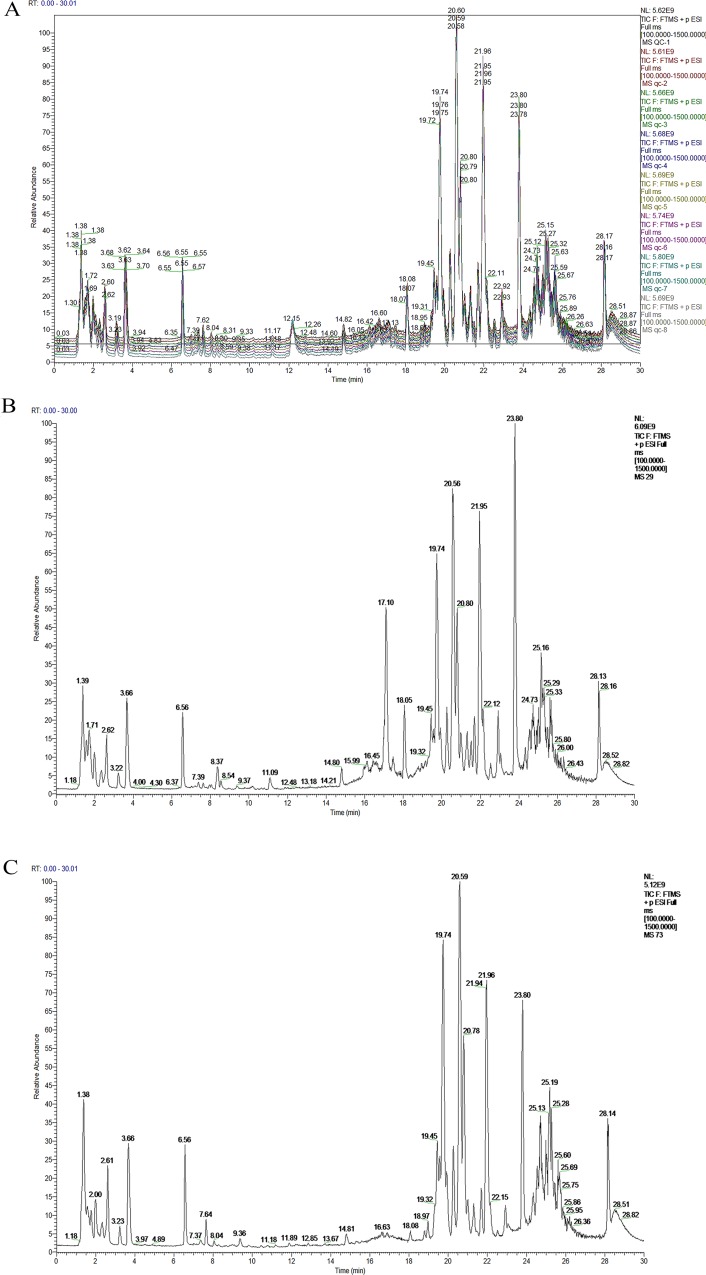
Typical LC-MS total ion chromatograms (TIC) from serum samples of the Quality Control (QC) sample (**A**), start point (**B**) and 1 week (**C**) sample.

**Figure 3:**
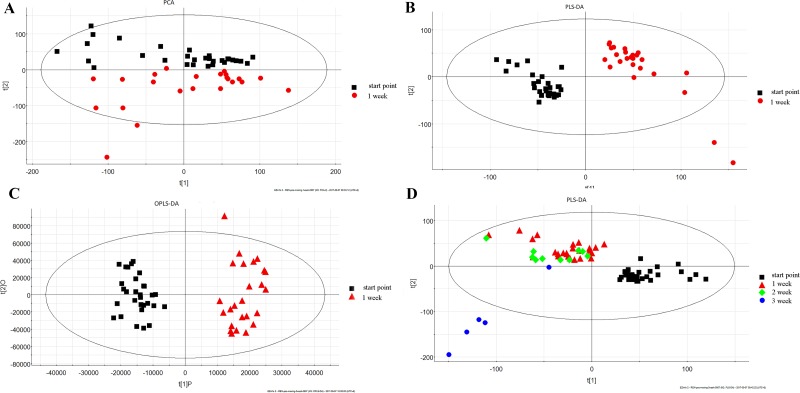
The score plots following (**A**) PCA, (**B**) PLS-DA, and (**C**) OPLS-DA analysis between start point and 1 week; (**D**) PLS-DA analyses of the different time.

The roadmap for experiment design was shown in Figure 1. To determine the analytical stability of LC-MS based methods for global serum metabolic profiling, a pooled quality control (QC) sample was repeatedly analyzed during sample runs. The overlapped total ion current (TIC) chromatograms of the QC sample demonstrated the strong repeatability of our LC-MS system (Figure 2A). Typical TIC chromatograms of the serum metabolic profiles of the start point and 1 week analyzed by LC-MS were shown in Figure 2B–2C. Serum metabonomics was used to assess differences between start point and 1 week. Principal component analysis (PCA) (Figure 3A) showed a spectral separation between these two groups, indicating significant metabolic differences. This was further supported by partial least squares-discriminate analysis (PLS-DA) (Figure 3B) and orthogonal partial least squares-discriminant analysis (OPLS-DA) (Figure 3C). The cumulative R^2^Y and Q^2^ for PLS-DA model were 0.95 and 0.84, respectively. The PLS-DA model was used to elucidate the most reliable separation of glucocorticoid injections groups at different time (Figure 3D). 39 metabolites were detected between start point and 1 week by OPLS-DA analysis, mainly included amino acid and phospholipid (Supplementary Table 1) when they met the following 3 criteria simultaneously: *P* < 0.05, CV < 0.3 and the top 50 of absolute value in the S-plot figure. The process of identification of metabolites were shown in Supplementary Materials.

**Figure 4:**
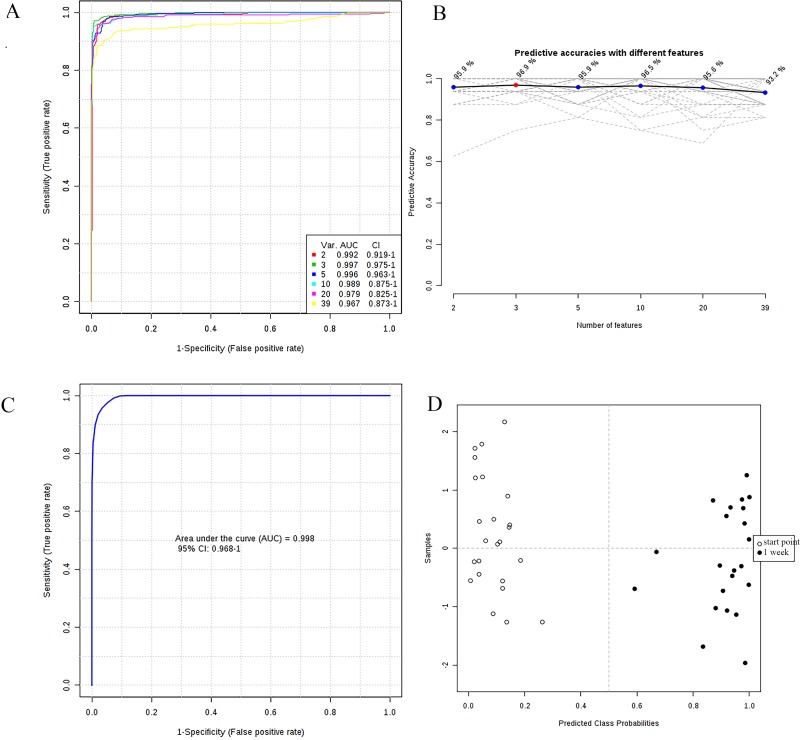
(**A**) ROC curves of all significant metabolites for 6 models; (**B**) Predictive accuracies with different models; (**C**) ROC curve by selecting metabolites; (**D**) Metabolic profiles depicted by score plots of new model by selecting metabolites.

We designed a prediction model and constructed receiver operating characteristic (ROC) curves to evaluate the accuracy of the model. Firstly, the data set was divided into builder and tester. The global performance of each biomarker model was evaluated using the area under the ROC curve (AUC). The cut-off point was defined by the minimum distance to the top-left corner using the builder samples. The sensitivity and specificity were determined at the optimal cut-off point. Then, PLS-DA was selected as classification method and Random Forest was selected as feature ranking method to construct the model. The AUC of most metabolites were satisfactory and the AUC of 34 of 39 metabolites were above 0.7. The ROC curves of 39 metabolites for 6 models were shown in Figure 4A. The model 2 (3 features) was optimal and the predictive accuracies was 96.9% (Figure 4B). Afterwards, metabolites with AUC larger than 0.7 was constructed on the builder samples to design the best metabolite combination by Random Forest algorithm. ROC curves were used to evaluate the accuracy of this model and predicted tester samples. The AUC of the constructed model was 0.998 (95% CI, 0.968–1) (Figure 4C). There was a visible separation in the score plots between start point and 1 week (Figure 4D). 3 samples of 1 week and 6 samples of start point were selected as tester. The predicted results on tester by the constructed model were great and 9 testers were classed to the appropriate group.

**Figure 5:**
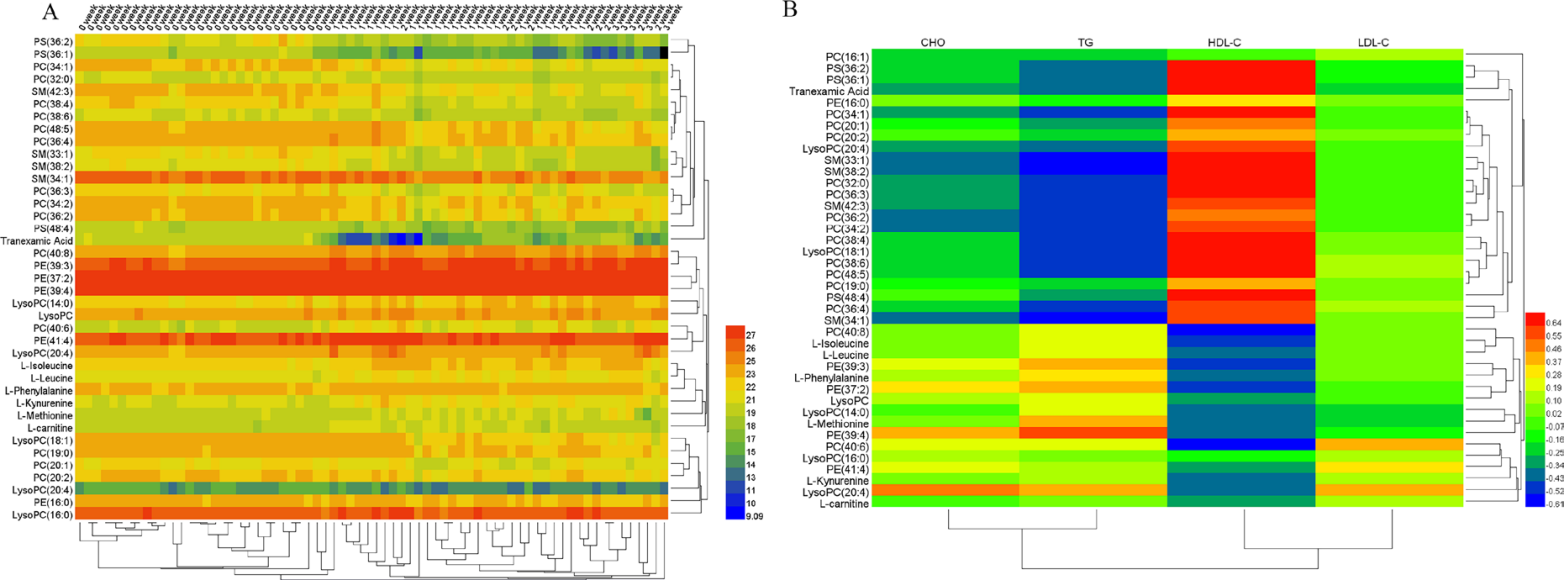
Heat map of the endogenous metabolites at different time (**A**) and heat map of correlation coefficient of the metabolites and biochemical index (**B**).

According to the peak area of each metabolite of four groups, the Heml 1.0.3.7 software packages were used to make a heat map in order to analyze these differential metabolites globally. The sample and metabolite differences were simultaneously hierarchically clustered. The horizontal axis of the figure showed a dendrogram of the samples. The samples of the different time were clustered together. The concentrations of different metabolites varied significantly at different time. The vertical axis of the figure showed a dendrogram of the metabolite differences. Significant clustering and relevance of the metabolites was shown in the heat map (Figure 5A). The metabolites in the same or similar metabolic pathways were clustered together first. The correlation coefficient of the metabolites and biochemical index was shown in Figure 5B. The high-density lipoprotein cholesterol (HDL-C) had the relative higher positive correlation with the most metabolites. The triglyceride (TG) had the higher negative correlation with the most metabolites.

**Figure 6:**
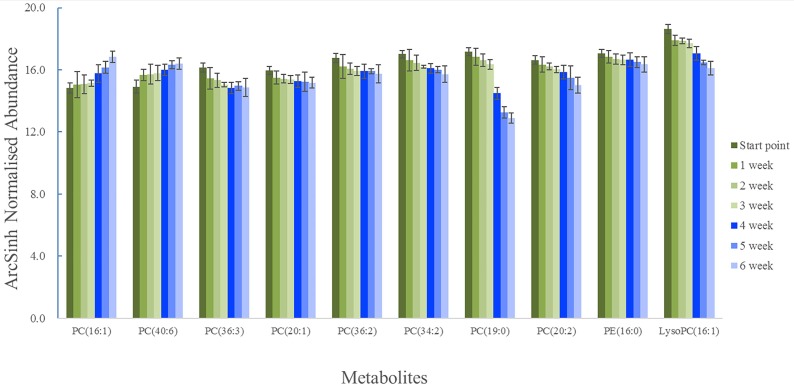
The trend of representative metabolomics of the different time

The arcsinh normalized abundance of representative metabolites at different time was shown in Figure 6. The normalized abundance of metabolites were transformed by arcsinh function because their distributions were skewed. The arcsinh was incremental function and it could describe the trend of contents of metabolites in every group. The green columns were first discovery experiment and the blue columns were second verified experiment. The contents of some metabolites (PC (36:3), PC (20:1), PC (36:2), PC (34:2), PC (19:0), PC (20:2), PE (16:0) and LysoPC (16:1)) of start point were significantly higher than other time (*P* < 0.05). The contents of some metabolites (PC (16:1) and PC (40:6)) of start point were significantly lower than other time (*P* < 0.05). At the second animal experiment, the research was design to verify the metabolites. These 10 compounds in 3–6 weeks had the same trend of the stage of 0–3 weeks.

**Figure 7:**
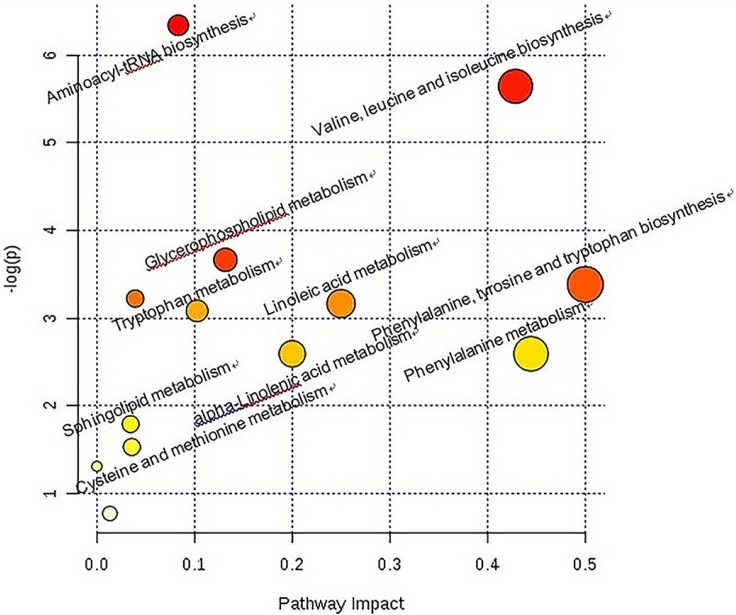
Construction of the altered metabolism pathways in glucocorticoid injections in Japanese white rabbits

To study the possible metabolic pathways after injecting glucocorticoid, we performed pathway analysis by metaboanalyst 3.0. The pathway analysis results showed the metabolic network with detailed impact (Figure 7). The influenced metabolic pathway was set as a pathway impact >0.05 and –log (*P*) > 2.50. The biological pathway analysis revealed eight main metabolic pathways, including aminoacyl-tRNA biosynthesis, valine, leucine and isoleucine biosynthesis, glycerophospholipid metabolism, phenylalanine, tyrosine and tryptophan biosynthesis, linoleic acid metabolism, tryptophan metabolism, phenylalanine metabolism, and alpha-linolenic acid metabolism.

**Figure 8:**
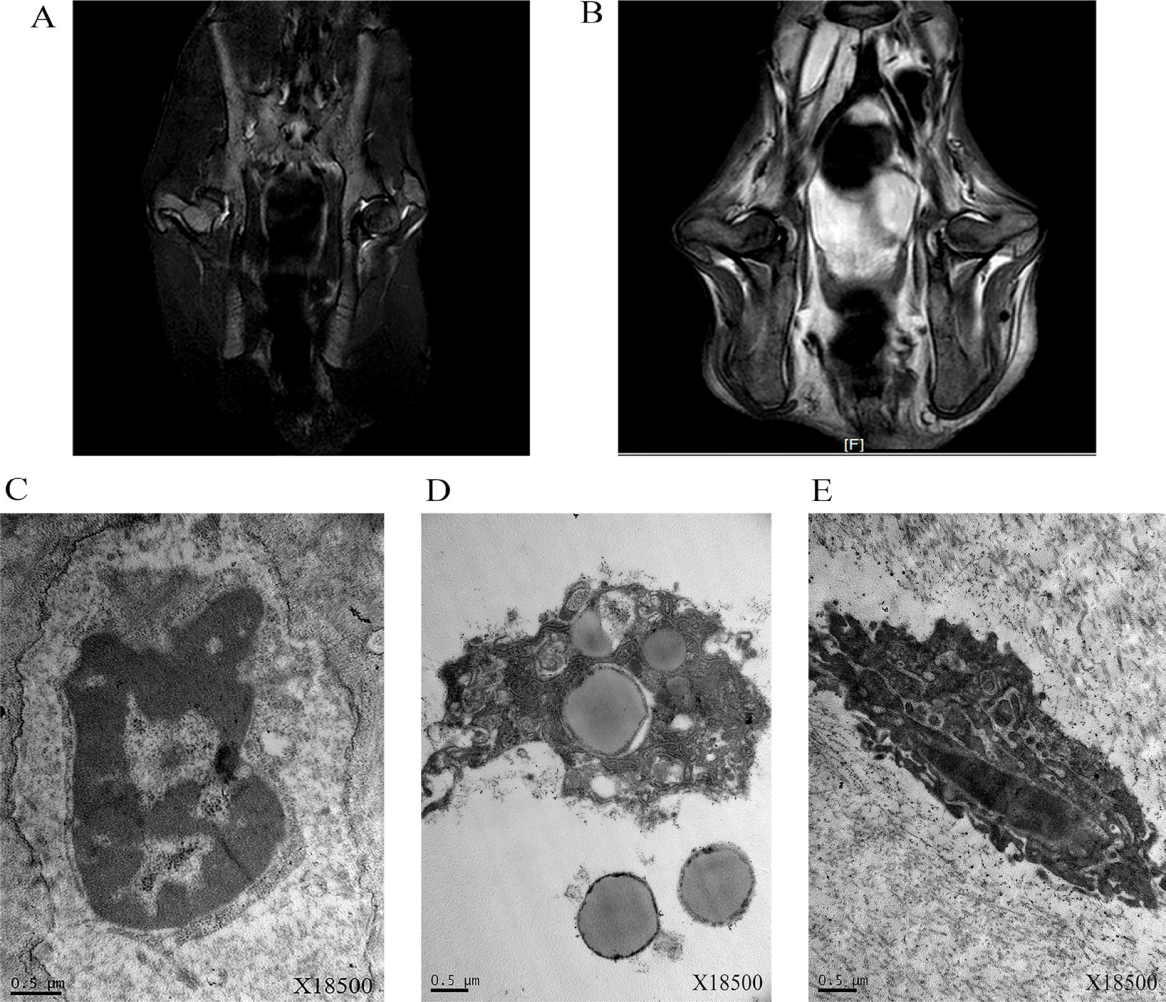
MRI (T_2_WI) of bilateral femoral heads of rabbits of start point (**A**) and 3 week (**B**) and transmission electron microscopy (TEM) imaging of bilateral femoral heads of rabbits of start point (**C**) and 6 week (**D**, **E**).

The results of magnetic resonance imaging (MRI) and transmission electron microscopy (TEM) were shown in Figure 8. The signal of femoral head was normal, and there was no abnormality inside and outside the joint soft tissue. The bilateral femoral heads slightly got collapsed and flattened 3 weeks later. There was obviously strip long T1 short T2 signal under femoral heads, and it was bilateral feature. The STRI sequence showed that femoral heads linear low signal and a little patchy hyperintensity round, the joint space widened slightly with a little amount of long T1 long T2 signal, and there was not obviously abnormal signal in the surrounding soft tissue. At the start of this study, TEM results revealed that chromatin was fine and the nucleolus was not obvious. The number and morphology of endoplasmic reticulum and mitochondria were normal. Lysosome could not be observed. After six weeks with injection of hormone, some cells contained hyperchromatic nuclei and exhibited irregular nuclear membrane and local fragmentation. The numbers of mitochondria, lysosome, and endoplasmic reticulum were increased. Some mitochondria swelled.

## DISCUSSION

SANFH has very diverse clinical and laboratory manifestations. However, the early diagnose of SANFH was challenging. At present, early prevention of SANFH depends on MRI scan, which was the standard of SANFH diagnose [7, 8, 30]. At the early stage of SANFH, blood supply was interdicted in the femoral head within 6–12 hours, which may lead to blood cell death. But the amount of these cells were less than fat cells and MRI may not reflect the lesion of femoral head. MRI scan only gave the osteonecrosis signal after fat cell death (12–48 hours), when some pathologic changes appeared, such as disorder of bone trabecula, pimelosis of marrow cavity, fatty degeneration and necrosis of osteocyte and so on [9]. Through MRI and observation on histopathology, we found that joint space of femoral heads got wide slightly and had a little patchy hyperintensity round 3 weeks later. More obviously changes, such as chromatin, nuclear membrane, nucleolus, lysosome, endoplasmic reticulum and mitochondria were discovered 6 weeks later, which were essential changes. Therefore, an early and accurate diagnosis of disease was crucial to monitor and intervene as early as possible.

In this study, 39 distinct metabolites and eight main metabolic pathways were revealed. The AUC of the optimized model was indicated the satisfactory sensitivity and specificity [31, 32]. Moreover, we performed an independent test experiment including 3 samples of 1 week and 6 sample of start point and the results showed that these 34 metabolites could differentiate samples of 1 week from start point, which indicated that the model had a high clinical value for the diagnosis of SANFH. Considering the AUC, *T*-test and fold change, 10 compounds of 39 metabolites were selected. After injecting glucocorticoid, the contents of phosphatidyl ethanolamine (PE) decreased and most of contents of phosphatidylcholine (PC) also decreased, which meant that most lipid anabolism was repressed. In order to prove whether or not 10 metabolites were relevant with the index of end point of SANFH, the verification test was carried out. Though verification, these 10 metabolites in the end point of verified experiment had the same trend of the discovered experiment. In other words, 10 compounds, mainly contained phosphatidylcholine (PC), were the representative metabolites and could be considered as the risk factor of steroid-induced femoral head necrosis, which were consistent with literature [4, 20]. The SANFH may be monitored using these 10 metabolites 1 week later when other indexes were not obvious, which may improve the effect of early prevention and cure of SANFH.

According to the heat map, the sample and metabolite differences were simultaneously hierarchically clustered. The metabolites between start point and 1 week were separated obviously and shown in the horizontal axis by HCA. The vertical axis of the figure showed a dendrogram of the metabolite differences and the metabolites from the same branch may be classed in the same class [33]. Additionally, some metabolisms were disrupted in rabbit models of SANFH. These metabolism alterations were related to disruption of the amino acid metabolism and lipid metabolism [18, 27]. Some researchers found that abnormal lipid metabolism is a strong risk factor, which may be the key pathomechanism of steroid-induced necrosis of femoral head [34].

On the other hand, serum metabolomics might provide a better understanding of potential metabolites that may differentiate start point from 1 week. After injecting glucocorticoid, the contents of PC (36:3), PC (20:1), PC (36:2), PC (34:2), PC (19:0), PC (20:2), PE (16:0) and LysoPC (16:1) were lower and the contents of PC (16:1) and PC (40:6) were higher than start point. These findings suggested the early metabolites changes were closely related to the phospholipid and lipoprotein. Lysophosphatidylcholines (C16:0 LPC, C18:0 LPC, C18:1 LPC and C18:2 LPC), tryptophane and phenylalanine, which were proved to be closely associated with osteoporosis, were identified in the rats plasma [20]. The osteoporosis was one of the hypothesis of SANFH [4]. Moreover, apoptosis was closely related to the femoral head necrosis [35, 36]. Tian Z. *et al.* [37] found that MiR-145 silencing promotes bone repair of ANFH via upregulating VEGF, bFGF and inhibiting the bone cells apoptosis through Wnt/β-catenin pathway. The apoptotic cells contained hyperchromatic nuclei and exhibited irregular nuclear membrane and local fragmentation, which were consistent with the change of pathology 6 weeks later. So after injecting the glucocorticoid, apoptosis was emerged. In the process of apoptosis, the permeability of cytomembrane gradually increased, and the transmembrane potential of mitochondria decreased [38]. The phospholipid was the basic shelf of the cytomembrane and they keep the cell stable. When the permeability of cytomembrane increased, the component of cytomembrane may change. It is difficult to keep cell stable for the less phospholipid. When the permeability of cytomembrane increased, the phospholipid may decreased. Just as the results of our study, the contents of most phosphatidylcholine decreased, which may be closely associated with the apoptosis.

Indeed, the identification and validation of biomarkers is one of the bottlenecks of current metabolomic studies. In this manuscript the finding of 10 metabolites relevant with early change of SANFH has been verified by a second animal experiment. These metabolites were identified as phospholipid family (PC or PE) according to their retention time, molecular and fragment ions, literatures and goodness of fitting. The subclass of phospholipid including number of carbon and double bonds have been identified, however, the accurate position of unsaturated bond are unknown. Therefore we have not validated using reference substance before their accurate position of unsaturated bond are determined since there are too many isomers for the phospholipid family while few of them is commercially available. Anyway it's one of the most important steps to identify the subclass of phospholipid including number of carbon and double bonds, and of course there is some emerging technology such as coupling the Paternò–Büchi (PB) reaction with infusion ESI-MS/MS for locating unsaturated bond locations in unsaturated lipids, which enable the determination of the accurate position of unsaturated bond [39, 40]. Moreover, the serum samples were collected from rabbits, which could provide the diagnose signals for SANFH. Further study was needed for the samples from clinical patients.

In conclusion, the early changes of SANFH by serum metabolomics and the potential biomarkers of SANFH at the early stage were studied. The diagnosed model was evaluated by ROC analysis and satisfactory verification results were obtained. Through verification, 10 metabolites, mainly contained phosphatidylcholine (PC), were closely associated with early changes of SANFH. The SANFH may be monitored using these 10 metabolites 1 week later when clinical symptom and imageological change were not obvious. The change of these 10 metabolites may be closely associated with the apoptosis and could be considered the risk factor of early prevention and intervention of SANFH.

## MATERIALS AND METHODS

### Chemical and reagents

Acetonitrile, methanol, isopropanol and formic acid were HPLC grade and purchased from Fisher (USA). Sodium pentobarbital solution was obtained from the Fourth Affiliated Hospital of Nanchang University. Prednisolone acetate injection was purchased from the company of Xianju pharmacy (Zhejiang, China). Benzylpenicillin sodium for injection was obtained from company of Huabei pharmacy (Shijiazhuang, China). Ultrapure water was purified by a Milli-Q system (Millipore, Bedford, MA, USA).

### Animal handling

Rabbit care and use were conducted in accordance with the recommendations in the Guide for the Care and Use of Laboratory Animals of the National Institutes of Health. All protocols were approved by the Medical Animal Studies Committee of the Fourth Affiliated Hospital of Nanchang University. A total of 35 healthy adult Japanese white rabbits for discovery were purchased from Animal Center of Tsinghua University. All animals were male, body weight 2.3–2.7 kg. All animals could eat and drink freely at a temperature of 20 **±** 3°C, and were fed in single cage. All animals were received the standard chew diet, and were kept on a cycle of 12 h light and 12 h dark, with the darkness starting from 19:00. After 1 week, the rabbits were weighed, gotten venous blood to analyze the biochemical index. Except the biochemical index anomaly and the accidental death, 30 healthy adult Japanese white rabbits were used for discovery experiment, which were injected prednisone solution. Except the accidental death and the killed rabbits, the residue rabbits were gotten venous blood 1 week, 2 weeks and 3 weeks later.

Moreover, a total of 15 healthy adult Japanese white rabbits for verification were purchased from Animal Center of the Fourth Affiliated Hospital of Nanchang University. The design was similar to the previous animal experiment, but it had the different sample collection time. Except the biochemical index anomaly and accidental death, 13 healthy adult Japanese white rabbits were used for discovery experiment, which were injected prednisone solution. Except the accidental death, the residue rabbits were gotten venous blood 3 weeks, 4 weeks, 5 weeks and 6 weeks later.

### Sample collection and preparation

At the sampling time, specific rabbits were tied to the laboratory table. The auricular skin was sterilized for a second time with 70% medical alcohol and above 3 mL venous blood was gotten. After the blood samples placed for 3 hours and centrifuged for 10 min (4°C, 3500 r/min). The supernatant was serum sample and was stored in a microtube and preserved at −80°C fridge.

Serum samples were taken out from −80°C freezer and were thawed over night at 4°C refrigerator. On the following day, after vortex 3 min by using Vortex Mixer XW-80A, serum sample (100 μL) was taken by pipette for metabolomics analysis. The proteins in sample were removed by using 400 μL acetonitrile. The samples were mixed for 3 min and centrifuged at 14480 × g for 10 min at 4°C in order to remove solid particles from the supernatant. The supernatant was evaporated to dryness by Centrivap Concentrator (LABCONCO, USA). The residue was reconstituted in the initial mobile phase, vortex 3 min and ultrasonic extracted 5 min at 4°C. The samples were centrifuged at 14480 × g for 20 min at 4°C twice and supernatant was transferred into 150 μL glass insert in a 1.5 mL amber glass vial and analyzed.

### Serum biochemistry

Serum biochemical analyses were performed on an automatic biochemistry analyzer (Cobas C501, Roche, Switzerland) including total cholesterol (CHO), high density lipoprotein cholesterol (HDL-C), low density lipoprotein cholesterol (LDL-C) and triglyceride (TG) levels. One-way ANOVA was conducted to analyze the clinical biochemical data from different groups.

### Magnetic resonance imaging scan and observation on histopathology

At starting point and 1 week, 2 weeks and 3 weeks later, MRI imaging was performed. When the rabbits were fastened, 35 mg/kg 1% sodium pentobarbital solution was injected into the ear vein. When anesthesia was effective, coronal scan of bilateral femoral heads of all rabbits was performed with Philips Achieva 3.0T TX MRI. The indexes were as follows: 3D T_2_WI VISTA (TR 2600 ms, TE 200 ms), slice thickness 1.2 mm, flip angle 90 deg and matrix (reconstruction 256 **×** matrix scan 256). Then the MRI results of early steroid-induced avascular necrosis of femoral head (SANFH) were compared with among the groups.

At starting point and 6 weeks later, transmission electron microscopy (TEM) imaging was used to observe the changes of histopathology. According to the protocol, the specimens were fixed, decalcified, wrapped, cut into slices and observed by TEM [41].

### UHPLC-MS/MS analysis

UHPLC-MS/MS analysis was performed using Q Exactive Plus High Resolution Mass Spectrometer (THERMO, USA) equipped with Ultimate 3000 (DIONEX, THERMO, USA). Acquity™ UPLC BEH C18 column (2.1 × 100 mm, 1.7 μm) (Waters, USA) was used. The optimized chromatographic conditions were achieved at a flow rate of 0.3 mL/min with a mobile phase consisting of isopropanol/facetonitrile 50:50 (V/V) with 0.1% formic acid (mobile phase A) and 0.1% formic acid solution (mobile phase B). The gradient elution process was: 0–2 min, 2% A; 2–22 min, 2–99% A; 22–25 min, 99% A; 25.1–30 min, 2% A. The injection volume was 10 μL. The sampling needle was washed with 300 μL of wash (methanol/water 50/50 V/V) once between two injections. The mass spectrometer was operating in full MS mode at a mass range of 100–1500 m/z in positive ionization.

### Statistical analysis and metabolite identification

The raw data were acquired using Xcalibur Qual Browser (THERMO, USA). The data were imported into the Progenesis QI (Waters, USA) for data analysis. Pre-treatment procedures including peak finding, alignment, filtering and normalization were performed to process the raw data. Principal component analysis (PCA) was used to obtain an overview of variations among the different groups. For the multivariate analysis, partial least squares-discriminate analysis (PLS-DA) and orthogonal partial least squares-discriminant analysis (OPLS-DA) were conducted. Finally, the OPLS-DA S-plot model was used to select key metabolites. Features with *P* < 0.05, CV<0.3 and the top 50 of absolute value in the S-plot figure were regarded as key metabolites and selected for identification. For LC/MS datasets, peaks were preliminarily identified by referring to online databases (HMDB, METLIN, Basic lipid, Glycerol lipid and Lipid MAPS). The results were matched with the molecular and fragment ions of experiment MS/MS spectra as well as confirmed with that of literatures [25, 26].

To validate the importance of the metabolites and to further evaluate their ability to distinguish between start point and 1 week, their potential predictive utility was assessed by receiver operating characteristic (ROC) curve analysis using MetaboAnalyst (http://www.metaboanalyst.ca/) [42]. ROC analysis was performed using MS peak areas corresponding to the metabolite concentrations in each group. The models built by the builders were used to predict response in the corresponding independent tester samples, and the area under the Receiver Operating Characteristic (ROC) curve (AUC), as well as the sensitivity and specificity were calculated to evaluate model performance for tester samples.

The Heml 1.0.3.7 software packages were used to make a heat map to represent the relative amount of differential metabolites and correlation coefficient of the metabolites and biochemical index, and to process their hierarchical cluster analysis (HCA) [43]. The pathway analysis of the dataset of identified key metabolites was also performed using MetaboAnalyst. The identified key metabolites at different time of glucocorticoid injections groups were shown in the histogram and were proceeded normality test and one-way ANOVA by SPSS software (Ver. 19.0) (IBM, USA). Differences were considered significant at *P* < 0.05.

### Quality control

For validating the stability of the analysis process, quality control sample (QC) was first prepared by mixing serum from each sample (10 μL). QC was injected ten times before the batch process. Water sample and QC were injected one time every ten samples during the analysis process, to monitor the stability of sample preparation and instrument.

## SUPPLEMENTARY MATERIALS FIGURES AND TABLES



## Abbreviations

SANFH: steroid-induced avascular necrosis of the femoral head
ultra-high performance liquid chromatography tandem mass spectrometry analysis (UHPLC-MS/MS
PCA: principal component analysis
PLS-DA: partial least squares-discriminate analysis
OPLS-DA: orthogonal partial least squares-discriminant analysis
ROC: receiver operating characteristic
SARS: severe acute respiratory syndromes
MRI: magnetic resonance imaging
QC: quality control
TIC: total ion current
HCA: hierarchical cluster analysis
CHO: cholesterol
TG: triglyceride
LDL-C: low-density lipoprotein cholesterol
HDL-C: high-density lipoprotein cholesterol
TEM: transmission electron microscopy
PC: phosphatidylcholine
Lyso-PC: lysophosphatidylcholine
PE: phosphatidyl ethanolamine
TAG: triacylglycerol
ACC: acetyl-CoA carboxylase
FAS: fatty acid synthase.
