# Supplementary material for: A metabolomic study on early detection of steroid-induced avascular necrosis of the femoral head
Source: Oncotarget. 2018 Jan 10;9(8):7984–95. doi: 10.18632/oncotarget.24150 (PMC5814275; doi:10.18632/oncotarget.24150)
Supplement: Supplementary file 1 [file oncotarget-09-7984-s001.pdf]

A metabolomic study on early detection of steroid-induced avascular necrosis of the femoral head

SUPPLEMENTARY MATERIALS

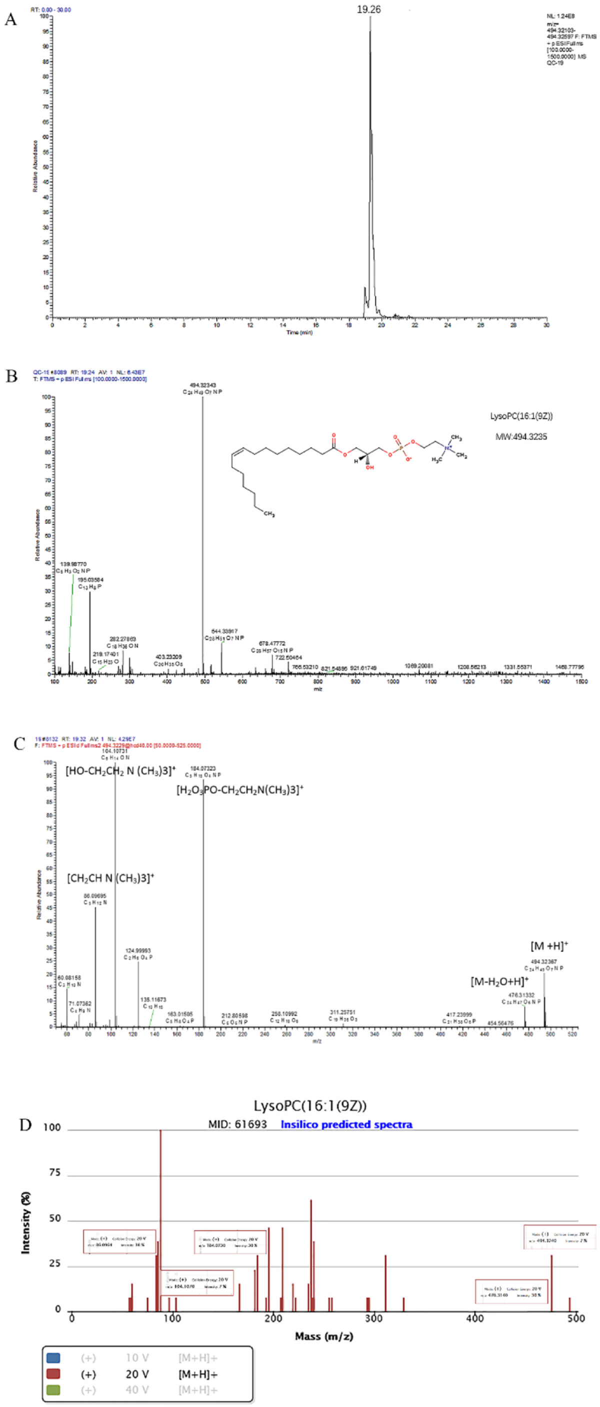

Supplementary Figure 1: Identification of a selected marker (**A**) Peak of potential biomarker of m/z 494 in extracted ion chromatogram with positive mode; (**B**) compound structure and corresponding mass spectrum; (**C**) MS/MS spectrum on the collision energy was 20 eV;

(**D**) standard MS/MS fragmentation in the metlin databases. We took the ion of m/z 494.3235 as an example to illustrate the biomarker identification process. Firstly, extracted ion m/z 494.3235 in chromatogram, the retention time 19.26 was obtained and was in accord with the measurement (Figure 1A). Secondly, the accurate molecular weight of the ion of $[M+H]^+$ was determined to be 494.3234 in the spectrum (Figure 1B). Thirdly, the assistant software packed in Xcalibur was used to determine the element composition for the peak at m/z494.3235. The calculated list provided several possible element compositions when considering the elements of carbon, hydrogen, nitrogen, oxygen and phosphorous (C, H, N, O and P). The lower the error value, the better the fit. After a series of analysis, only one possible element composition of $C_{24}H_{49}NO_7P$ was obtained (Figure 1B). Fourthly, the element composition was compared to those registered in the databases, and the metabolite was preliminarily identified as LysoPC (16:1). Lastly, mass fragmentation experiment was conducted to confirm the identification. The fragments of m/z 86, 104,184 and 476 were observed in MS/MS spectrum (Figure 1C), where m/z 86.0970 stands for $[CH_2CHN\ (CH_3)3]^+$, m/z 104.1074 stands for $[HOCH_2CH_2N\ (CH_3)3]^+$, 184.0734 stands for $[H_2O_3PO\text{-}CH_2CH_2N\ (CH_3)3]^+$, both of them are typical fragments of PC, and moreover, 476.3140 stands for $[M\text{-}H_2O+H]^+$, further supporting the suggestion that this metabolite belongs to the class of LysoPC, all these data showed very good accordance with standard spectrum in the metlin databases (Figure 1D). Based on all the information we got from the above process, the biomarker was identified as LysoPC (16:1).

Supplementary Table 1: Serum metabolites differentially expressed in glucocorticoid injections groups at different time

	Compounds	m/z	RT (min)	0 week[a] (n = 25)	1 week[b] (n = 25)	2 week[c] (n = 10)	3 week[d] (n = 6)
1	PC (16:1)	480.34	20.98	14.83 (0.33)	15.05 (0.84)	15.07 (0.59)	15.14 (0.21)
2	PS (36:2)	774.56	24.63	15.76 (0.59)	13.83 (0.58)	13.64 (0.52)	13.20 (0.44)
3	PS (36:1)	776.58	25.07	14.39 (0.58)	11.75 (1.04)	10.83 (0.92)	8.01 (4.04)
4	PC (34:1)	746.60	26.02	16.71 (0.24)	15.90 (0.53)	15.68 (0.35)	15.48 (0.04)
5	PS (38:4)	798.56	24.48	14.61 (0.41)	13.50 (0.58)	13.48 (0.50)	13.19 (0.32)
6	Tranexamic Acid	158.12	1.50	14.53 (0.55)	11.10 (1.70)	12.23 (2.04)	13.35 (1.00)
7	PC (40:8)	830.57	24.38	17.37 (0.34)	18.05 (0.36)	18.28 (0.27)	18.09 (0.27)
8	SM (33:1)	689.56	25.07	16.10 (0.27)	15.22 (0.64)	14.71 (0.74)	14.64 (0.30)
9	PC (32:0)	720.59	26.09	15.28 (0.26)	14.55 (0.55)	14.30 (0.37)	14.31 (0.13)
10	PC (38:4)	796.62	25.84	16.26 (0.29)	15.39 (0.63)	15.23 (0.42)	15.02 (0.38)
11	PC (38:6)	792.59	24.84	15.32 (0.34)	14.51 (0.63)	14.14 (0.57)	13.93 (0.36)
12	SM (38:2)	757.62	25.92	15.72 (0.23)	14.80 (0.76)	14.32 (0.69)	14.36 (0.35)
13	PC (48:5)	794.60	25.27	17.36 (0.33)	16.63 (0.59)	16.29 (0.53)	16.01 (0.29)
14	PE (39:3)	806.57	24.55	19.13 (0.23)	19.48 (0.23)	19.61 (0.14)	19.52 (0.13)
15	SM (42:3)	811.67	26.38	16.78 (0.30)	16.04 (0.69)	15.69 (0.45)	15.74 (0.23)
16	L-Isoleucine	132.10	2.56	16.14 (0.19)	16.59 (0.19)	16.53 (0.46)	16.73 (0.30)
17	LysoPC (18:1)	508.38	21.35	17.15 (0.23)	16.58 (0.61)	16.25 (0.44)	16.07 (0.22)
18	PC (36:4)	768.59	25.31	17.42 (0.29)	16.84 (0.53)	16.61 (0.49)	16.39 (0.24)
19	PC (40:6)	834.60	25.26	14.91 (0.43)	15.68 (0.36)	15.72 (0.65)	15.80 (0.49)
20	PC (36:3)	770.60	25.45	16.14 (0.28)	15.44 (0.69)	15.33 (0.46)	15.05 (0.14)
21	L-Phenylalanine	166.09	3.63	16.68 (0.15)	17.04 (0.17)	16.90 (0.34)	16.97 (0.31)
22	PC (20:1)	550.39	22.03	15.96 (0.26)	15.50 (0.41)	15.43 (0.27)	15.37 (0.26)
23	L-Kynurenine	209.09	3.19	15.34 (0.27)	15.90 (0.38)	15.84 (0.45)	15.72 (0.31)
24	L-carnitine	162.11	1.39	14.46 (0.27)	15.18 (0.48)	14.73 (0.61)	14.62 (0.39)
25	SM (34:1)	703.57	25.37	18.80 (0.16)	18.45 (0.44)	18.14 (0.49)	18.20 (0.20)
26	L-Leucine	132.10	2.33	15.60 (0.19)	15.95 (0.18)	15.86 (0.38)	15.91 (0.27)
27	LysoPC (20:4)	544.34	19.32	17.17 (0.31)	17.74 (0.52)	17.72 (0.59)	18.05 (0.44)
28	LysoPC (14:0)	468.31	18.95	16.14 (0.26)	16.58 (0.51)	16.78 (0.41)	16.90 (0.45)
29	PC (36:2)	772.62	26.05	16.77 (0.29)	16.22 (0.77)	16.07 (0.38)	15.92 ()0.29
30	PE (37:2)	758.57	25.15	20.52 (0.14)	20.67 (0.17)	20.75 (0.13)	20.82 (0.14)
31	PC (34:2)	744.59	25.52	17.01 (0.25)	16.61 (0.69)	16.45 (0.48)	16.20 (0.09)
32	PC (19:0)	538.39	22.52	17.17 (0.26)	16.84 (0.55)	16.63 (0.40)	16.35 (0.31)
33	PE (39:4)	782.57	24.70	19.75 (0.15)	19.98 (0.29)	20.08 (0.19)	20.04 (0.26)
34	L-Methionine	150.06	1.77	14.65 (0.25)	15.25 (0.29)	14.77 (0.70)	15.01 (1.42)
35	PC (20:2)	548.37	21.04	16.64 (0.28)	16.34 (0.48)	16.22 (0.22)	16.02 (0.20)
36	PE (16:0)	454.29	20.77	17.06 (0.25)	16.85 (0.39)	16.70 (0.34)	16.64 (0.31)
37	LysoPC (16:1)	494.32	19.28	18.62 (0.29)	17.90 (0.34)	17.88 (0.18)	17.71 (0.27)
38	PE (41:4)	810.60	25.46	19.31 (0.22)	19.54 (0.24)	19.53 (0.39)	19.42 (0.23)
39	LysoPC (16:0)	496.34	20.25	19.08 (0.17)	19.18 (0.29)	19.18 (0.21)	19.03 (0.22)

Note: 1 The data of the column of a-d was the arcsinh normalized abundance of metabolites.
2 The data was shown as mean value (standard deviation).
